# Supplementary material for: Sequential cocatalyst decoration on BaTaO2N towards highly-active Z-scheme water splitting
Source: Nat Commun. 2021 Feb 12;12:1005. doi: 10.1038/s41467-021-21284-3 (PMC7881033; doi:10.1038/s41467-021-21284-3)
Supplement: Supplementary file 1 — Supplementary Information [file 41467_2021_21284_MOESM1_ESM.pdf]

Electronic Supplementary Information (ESI)

Sequential cocatalyst decoration on $BaTaO_2N$ towards highly-active Z-scheme water splitting

List of the authors

Zheng Wang[1,2,9], Ying Luo[3,9], Takashi Hisatomi[1], Junie Jhon M. Vequizo[1], Sayaka Suzuki[4], Shanshan Chen[1], Mamiko Nakabayashi[5], Lihua Lin[1], Zhenhua Pan[1], Nobuko Kariya[6], Akira Yamakata[7], Naoya Shibata[5], Tsuyoshi Takata[1], Katsuya Teshima[1,4*] and Kazunari Domen[1,8,*]

Affiliation and full postal address

1. Research Initiative for Supra-Materials, Interdisciplinary Cluster for Cutting Edge Research, Shinshu University, 4-17-1 Wakasato, Nagano-shi, Nagano 380-8553, Japan
2. Research Center for Eco-Environmental Sciences, Chinese Academy of Sciences, Beijing 100085, China
3. Department of Science and Technology, Graduate School of Medicine, Science and Technology, Shinshu University, 4-17-1 Wakasato, Nagano 380-8553, Japan
4. Department of Materials Chemistry, Faculty of Engineering, Shinshu University, 4-17-1 Wakasato, Nagano 380-8553, Japan
5. Institute of Engineering Innovation, The University of Tokyo, 2-11-16 Yayoi, Bunkyo-ku, Tokyo 113-8656, Japan
6. Science & Innovation Center, Mitsubishi Chemical Corporation, 1000 Kamoshida-cho, Aoba-ku, Yokohama-shi, Kanagawa 227-8502, Japan

7. Graduate School of Engineering, Toyota Technological Institute, 2-12-1 Hisakata, Tempaku, Nagoya 468-8511, Japan
8. Office of University Professors, The University of Tokyo, 2-11-16 Yayoi, Bunkyo-ku, Tokyo 113-8656, Japan
9. These authors contributed equally: Zheng Wang and Ying Luo

* E-mail: domen@shinshu-u.ac.jp; teshima@shinshu-u.ac.jp

Supplementary Table S1 | Representative Z-scheme water splitting systems

HEP[a]	OEP[b]	Electron mediator	Reaction solution	Gas evolution rates[c] (μmol h^{-1})	Efficiency[d]
$Pt/SrTiO_3:Cr/Ta$[1]	PtO_x/WO_3	IO_3^-/I^-	H_2O	H_2: 1.8<br>O_2: 0.9	AQY: 0.1% at 420 nm
$Ru/SrTiO_3:Rh$[2]<br>(BG = 2.3 eV)	$BiVO_4$	Fe^{3+}/Fe^{2+}	H_2SO_4 solution<br>pH = 2.4	H_2: 122<br>O_2: 61	AQY: 4.2% at 420 nm;<br>STH: 0.1%
$Ru/SrTiO_3:La,Rh$[3]<br>(BG = 2.4 eV)	$RuO_2/BiVO_4:Mo$	Au layer	H_2O	H_2: 105<br>O_2: 52	AQY 33% at 419 nm;<br>STH: 1.1%
Pt/TaON[4]<br>(BG = 2.5 eV)	PtO_x/WO_3	IO_3^-/I^-	H_2O	H_2: 24<br>O_2: 12	AQY: 0.4% at 420 nm
$Pt/ZrO_2/TaON$[5]<br>(BG = 2.5 eV)	PtO_x/WO_3	IO_3^-/I^-	H_2O	H_2: 32.6<br>O_2: 15.6	AQY: 6.3% at 420 nm
$Pt/MgTa_2O_{6-x}N_y/TaON$[6]<br>(BG = 2.4 eV)	PtO_x/WO_3	IO_3^-/I^-	H_2O	H_2:108.3<br>O_2: 55.3	AQY: 6.8% at 420 nm
$Rh_yCr_{2-y}O_3/ZrO_2/TaON$[7]<br>(BG = 2.4 eV)	$Au/CoO_x/BiVO_4$	$Fe(CN)_6^{3-/4-}$	Na_3PO_4 solution<br>pH = 6.0	H_2: 130<br>O_2: 65	AQY: 10.3% at 420 nm
$Pt/IrO_2/Sm_2Ti_2S_2O_5$[8]<br>(BG = 2.25 eV)	PtO_x/H-Cs-WO_3	I_3^-/I^-	H_2O	H_2: 4.1<br>O_2: 1.6	STH: 0.003%
$Cr_2O_3/Rh/La_5Ti_2Cu_{0.9}Ag_{0.1}S_5O_7:Ga$[9]<br>(BG = 1.88 eV)	$BiVO_4$	Au layer	H_2O	H_2: 22<br>O_2: 11	AQY: 3.2% at 420 nm;<br>STH: 0.11%

Pt/$CuGaS_2$[10]<br>(BG = 2.3 eV)	$CoO_x/BiVO_4$	Reduced graphene oxide	H_2O	H_2: 3.5<br>O_2: 1.7	NR
Cr_2O_3/Pt/$(ZnSe)_{0.5}(CuGa_{2.5}Se_{4.25})_{0.5}$[11]<br>(BG = 1.79 eV)	$CoO_x/BiVO_4$	Au layer	H_2O	H_2: 3.8<br>O_2: 1.9	AQY: 0.54% at 420 nm;<br>STH: 0.01%
Pt/$H_4Nb_6O_{17}$ sensitized with Dye[12]<br>(Full visible light absorption)	$PtO_x/IrO_2/WO_3$	IO_3^-/I^-	H_2O	H_2: 1.8<br>O_2: 0.9	NR
Pt/$BaTaO_2N$[13]<br>(BG = 1.8 eV)	PtO_x/WO_3	IO_3^-/I^-	H_2O	H_2: 6.8<br>O_2: 3.2	AQY: 0.1% at 420−440 nm
Pt/$BaZrO_3$-$BaTaO_2N$ solid solution[14]<br>(BG = 1.8 eV)	PtO_x/WO_3	IO_3^-/I^-	H_2O	H_2: 5.8<br>O_2: 2.5	STH: 0.0067%
Pt/$BaTaO_2N/Ta_3N_5$ heterostructure[15]<br>(BG = 2.1 eV)	PtO_x/WO_3	IO_3^-/I^-	H_2O	H_2: 3.2<br>O_2: 1.6	AQY: 0.1% at 420 nm
Pt/$Ta_3N_5/BaTaO_2N$ heterostructure[16]<br>(BG = 2.1 eV)	PtO_x/WO_3	IO_3^-/I^-	H_2O	H_2: 4.8<br>O_2: 2.4	NR
Pt/$BaTaO_2N$ nitrided with flux[17]<br>(BG = 1.94 eV)	PtO_x/WO_3	IO_3^-/I^-	H_2O	H_2: 3.1<br>O_2: 1.6	AQY: 0.06% at 420 nm
Pt/$BaZrO_3$-$BaTaO_2N$ solid solution[18]<br>(BG = 1.8 eV)	PtO_x/WO_3	IO_3^-/I^-	H_2O	H_2: 5.5<br>O_2: 2.7	AQY: 0.6% at 420−440 nm

[a] HEP: H_2-evolution photocatalyst. [b] O_2-evolution photocatalyst. [c] Light source: 300 W Xe lamp $\lambda \geq 420$ nm. [d] AQY: Apparent quantum yield; STH: Solar-to-hydrogen energy conversion efficiency; NR: Not reported.

Supplementary Table S2 | Representative photocatalytic half-reactions of $BaTaO_2N$ photocatalysts.

Photocatalyst materials	Cocatalyst modification	Reaction solution	Gas evolution rates[a] (μmol h^{-1})	Efficiency[b]
$BaTaO_2N$[13] (BG = 1.8 eV)	Pt	Methanol solution	H_2: 50	NR
Ta_3N_5/$BaTaO_2N$ heterostructure[16] (BG = 2.1 eV)	Pt	Methanol solution	H_2: 27	NR
Flux-assisted $BaTaO_2N$[17] (BG = 1.94 eV)	Pt	Methanol solution	H_2: 16	NR
$BaZrO_3$-$BaTaO_2N$ solid solution[19] (BG = 1.8 eV)	Pt	Methanol solution	H_2: 14	AQY: 0.06% at 420 nm
Flux-assisted $BaTaO_2N$[20] (BG = 1.8 eV)	Pt	Methanol solution	H_2: 4.	NR
$BaZrO_3$-$BaTaO_2N$ solid solution[19] (BG = 1.8 eV)	IrO_2	$AgNO_3$ solution	O_2: 8.	AQY: 0.03% at 420 nm
$BaTaO_2N$:Mg[21] (BG = 1.88 eV)	CoO_x	$AgNO_3$ solution	O_2: 40	AQY: 2.6% at 420 nm
Flux-assisted $BaTaO_2N$[22] (BG = 1.8 eV)	CoO_x	$AgNO_3$ solution	O_2: 699	AQY: 11.9% at 420 nm

[a] Light source: 300 W Xe lamp $\lambda \geq 420$ nm. [b] AQY: Apparent quantum yield; NR: Not reported.

Electronic Supplementary Information (ESI)

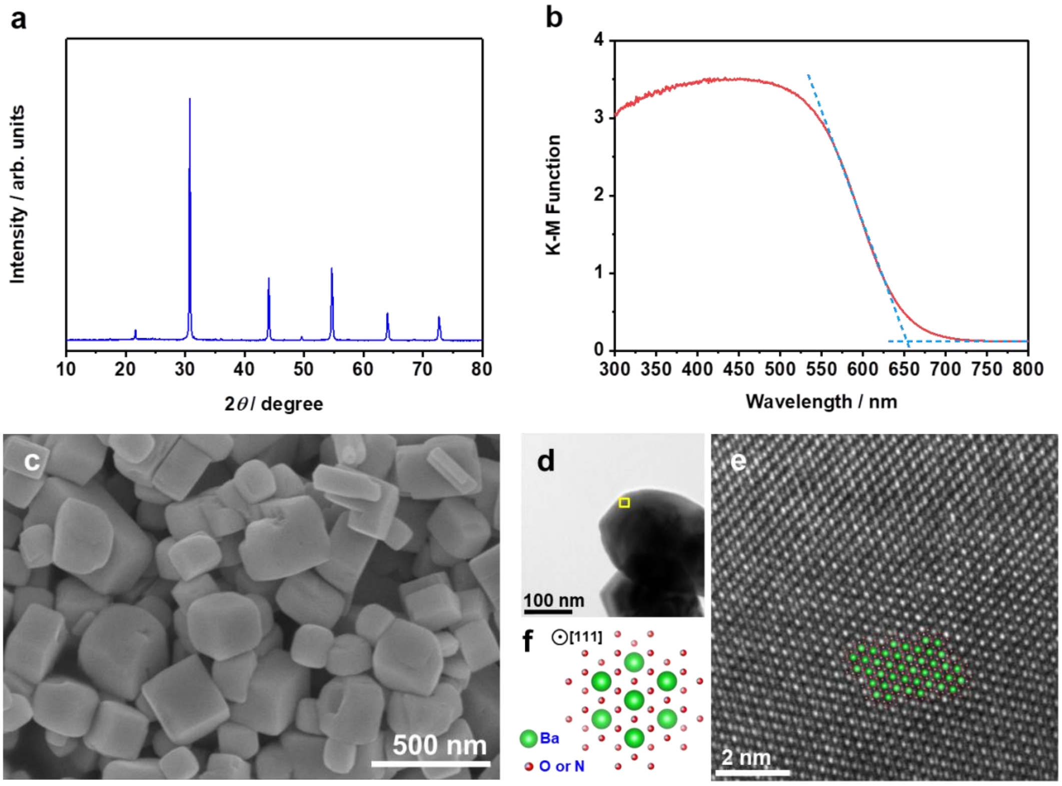

Supplementary Figure S1 | Characterization of pristine $BaTaO_2N$ (RbCl). a, XRD patterns. **b**, UV-vis DRS. **c**, SEM image. **d**,**e**, HRTEM images. **e** corresponds to the area marked with a square in **d**. Inset: crystal structure of $BaTaO_2N$ along the [111] direction, as shown in **f**. **f**, Crystal structure of $BaTaO_2N$ along the [111] direction, depicted using the Vesta programme[23].

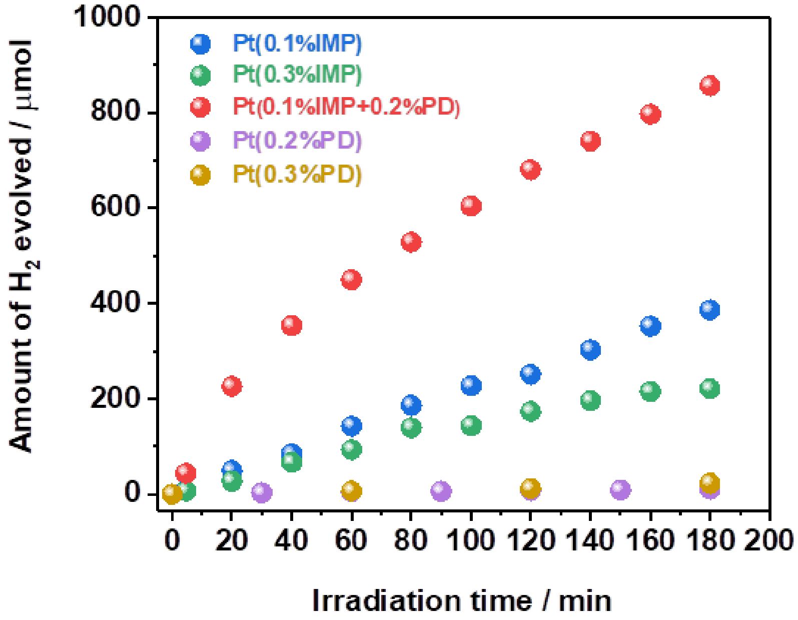

Supplementary Figure S2 | Photocatalytic H_2-evolution performance of Pt-modified $BaTaO_2N$ (RbCl). Time courses of photocatalytic H_2 evolution on Pt-modified $BaTaO_2N$ produced by three different methods. IMP, PD and IMP+PD denote Pt loading by impregnation-reduction, photodeposition, and sequential decoration, respectively. Conditions: Pt-modified $BaTaO_2N$ (RbCl), 0.1 g; 10 vol% aqueous methanol solution, 150 mL; light source, 300 W Xenon lamp ($\lambda \geq 420$ nm); reaction system, Pyrex top-illuminated vessel connected to closed gas-circulation system without evacuation of gas products.

Electronic Supplementary Information (ESI)

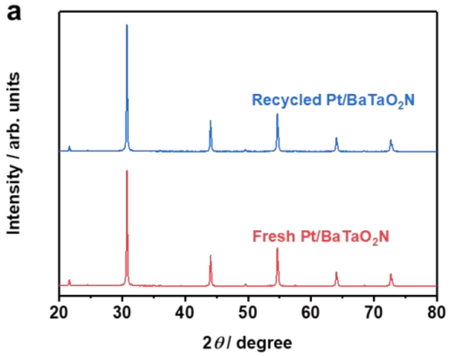

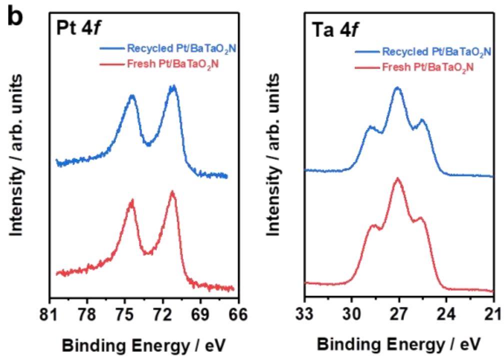

Sample	Atomic ratio between Pt and Ta by XPS Analysis (%)
Fresh Pt/$BaTaO_2N$	7.4
Recycled Pt/$BaTaO_2N$	8.0

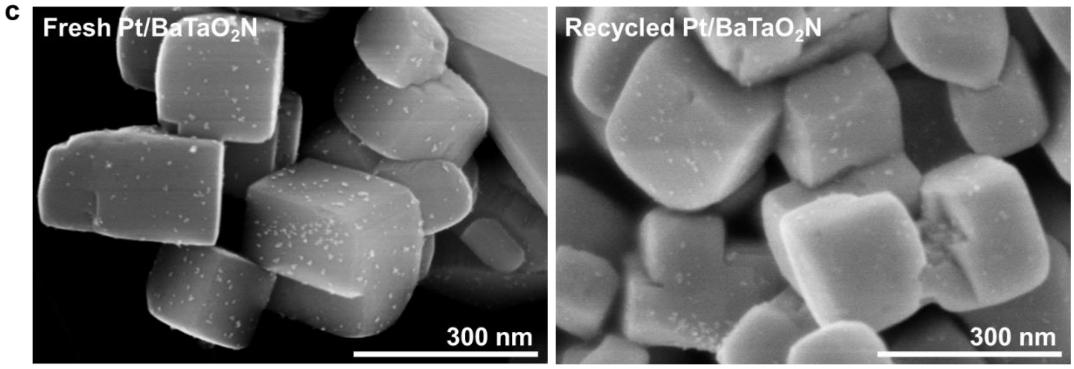

Supplementary Figure S3 | Characterizations of recycled Pt-modified $BaTaO_2N$ (RbCl) photocatalyst. a,**b**,**c**, XRD patterns (**a**), XPS spectra (Pt 4*f* and Ta 4*f*) (**b**) and SEM images (**c**) acquired from fresh and recycled Pt-modified $BaTaO_2N$ (RbCl) used in photocatalytic H_2 evolution reaction from methanol solution. Pt content was 0.3 wt% in total by sequential decoration method (0.1 wt% by impregnation-reduction and 0.2 wt% by photodeposition).

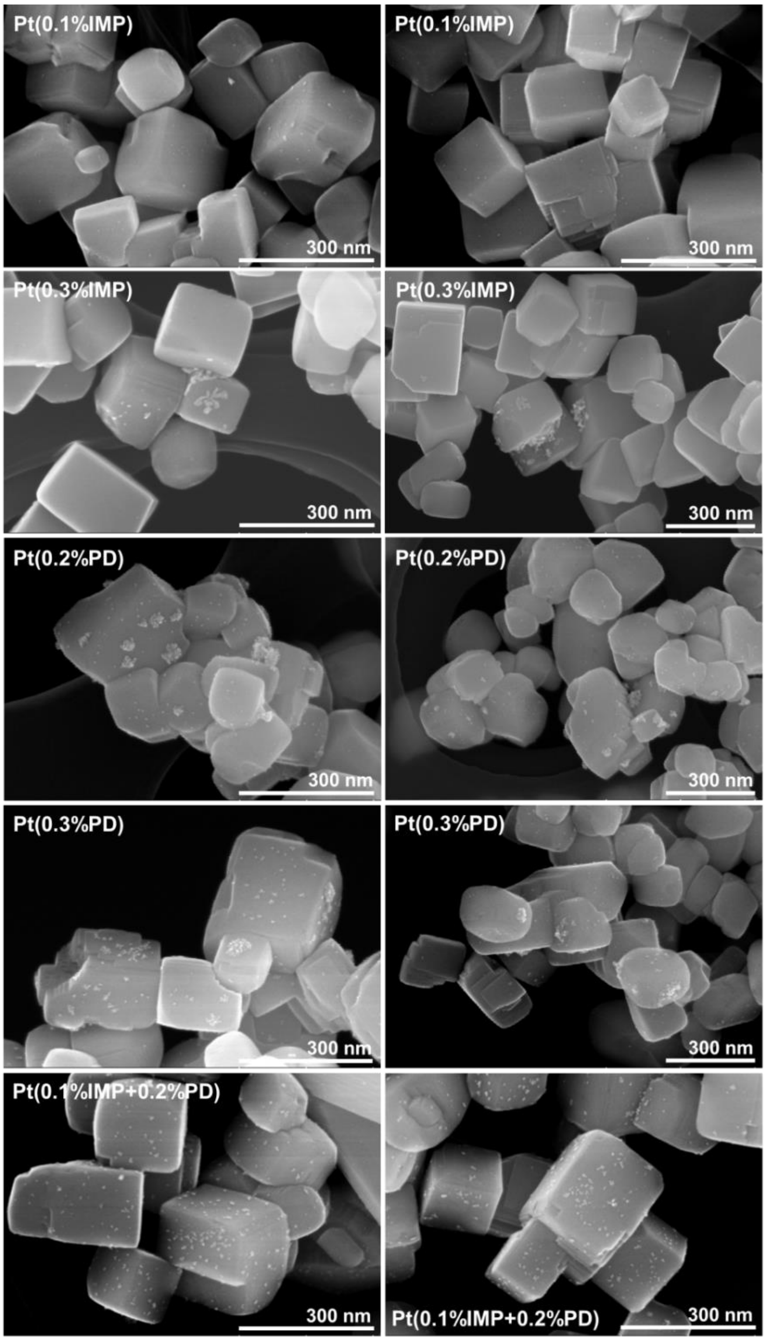

Supplementary Figure S4 | Morphology of Pt-modified $BaTaO_2N$ (RbCl). SEM images of Pt-modified $BaTaO_2N$ produced by three different methods. IMP, PD and IMP+PD denote Pt loading by impregnation-reduction, photodeposition, and sequential decoration, respectively.

Supplementary Table S3 | Mass ratio of total Pt cocatalyst in Pt-modified $BaTaO_2N$ photocatalysts analysed by ICP-AES.

Samples[a]	Pt contents / wt%
Pt(0.1%IMP)/$BaTaO_2N$	0.09
Pt(0.3%IMP)/$BaTaO_2N$	0.30
Pt(0.2%PD)/$BaTaO_2N$	0.23
Pt(0.3%PD)/$BaTaO_2N$	0.34
Pt(0.1%IMP+0.2%PD)/$BaTaO_2N$	0.31

[a] $BaTaO_2N$ was synthesized with RbCl flux.

Electronic Supplementary Information (ESI)

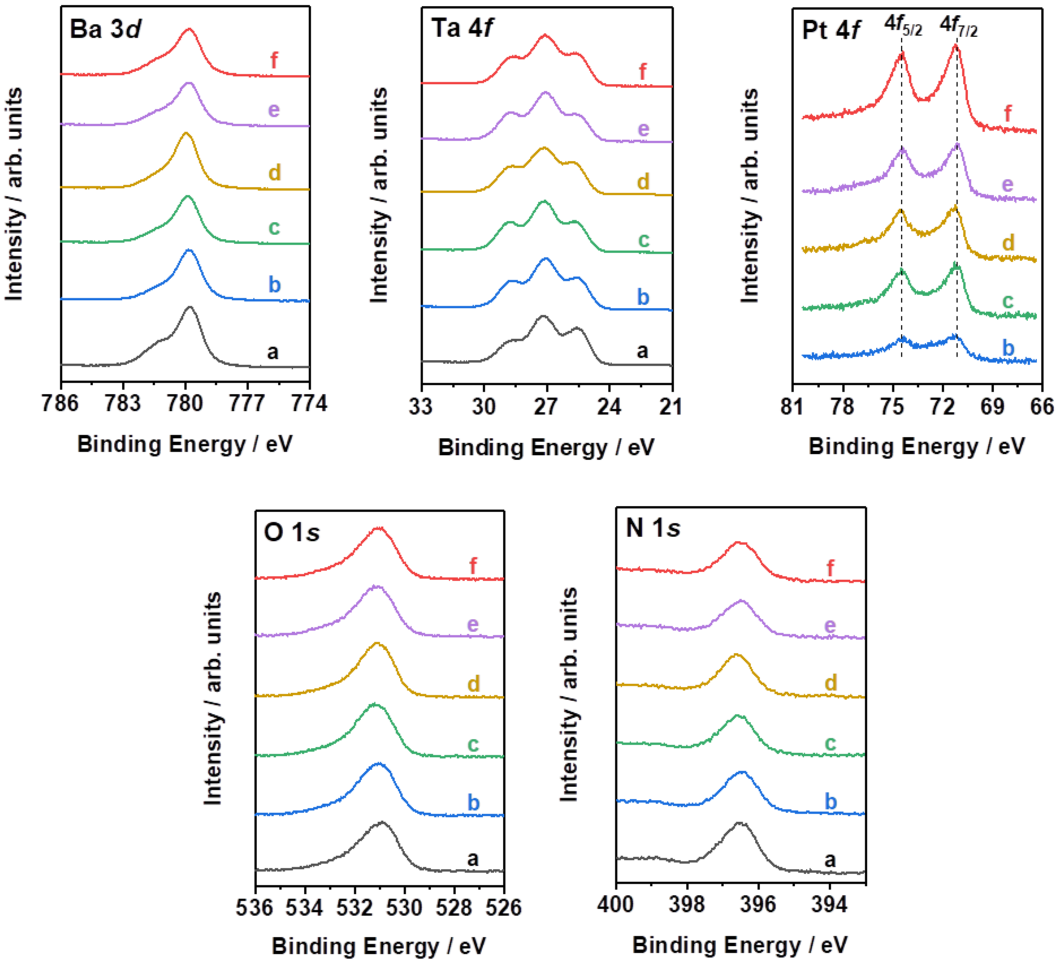

Supplementary Figure S5 | XPS spectra of pristine $BaTaO_2N$ (RbCl) and Pt-modified $BaTaO_2N$ (RbCl). a-f, Ba 3*d*, Ta 4*f*, O 1*s*, N 1*s* and Pt 4*f* spectra acquired from pristine $BaTaO_2N$ (**a**), Pt(0.1%IMP)/$BaTaO_2N$ (**b**), Pt(0.3%IMP)/$BaTaO_2N$ (**c**), Pt(0.2%PD)/$BaTaO_2N$ (**d**), Pt(0.3%PD)/$BaTaO_2N$ (**e**) and Pt(0.1%IMP+0.2%PD)/$BaTaO_2N$ (**f**).

The Pt $4f_{7/2}$ peaks for all the Pt-modified $BaTaO_2N$ samples were located at the binding energy for metallic Pt ($4f_{7/2}$: 71.2 eV)[24], indicating that the Pt cocatalysts deposited by the three methods were metallic. The peak position and intensity for Ba 3*d*, Ta 4*f*, O 1*s*

and N 1*s* for Pt-modified $BaTaO_2N$ samples were also in line with those for pristine $BaTaO_2N$. The surface components of the $BaTaO_2N$ photocatalysts remained unchanged after Pt modification by the different methods and did not influence the photocatalytic activity.

Electronic Supplementary Information (ESI)

Supplementary Table S4 | Estimation of remaining photoexcited electrons in pristine $BaTaO_2N$ and Pt-modified $BaTaO_2N$ photocatalysts based on the transient absorption at 300 μs.

Samples[a]	Absorbance at 300 μs[b]	Percentage of remaining electrons at 300 μs[c]
Pristine $BaTaO_2N$	4.5×10^{-5}	100%
Pt(0.1%IMP)/$BaTaO_2N$	2.0×10^{-5}	45%
Pt(0.3%IMP)/$BaTaO_2N$	2.9×10^{-5}	64%
Pt(0.2%PD)/$BaTaO_2N$	2.8×10^{-5}	63%
Pt(0.3%PD)/$BaTaO_2N$	4.2×10^{-5}	94%
Pt(0.1%IMP+0.2%PD)/$BaTaO_2N$	9.0×10^{-6}	20%

[a] $BaTaO_2N$ was synthesized with RbCl flux.

[b] Absorbance at 300 μs was acquired from the decay of transient absorption in Fig. 3a.

[c] The percentage of remaining electrons in each samples was calculated with respect to that in bare $BaTaO_2N$.

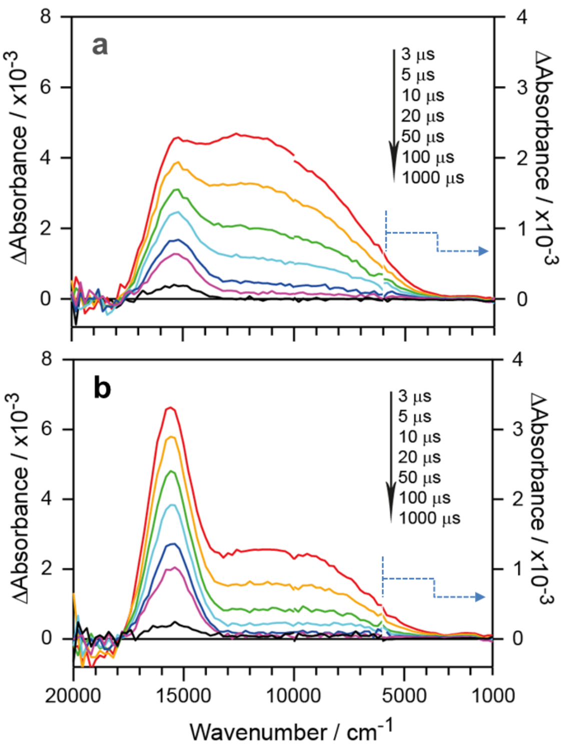

Supplementary Figure S6 | TA measurements of pristine $BaTaO_2N$ (RbCl) and Pt-modified $BaTaO_2N$ (RbCl). a,**b**, Time-resolved TA spectra of pristine $BaTaO_2N$ (**a**) and Pt(0.1%IMP+0.2%PD)/$BaTaO_2N$ (**b**). The left axis indicates the absorbance in the visible to NIR region from 20000 cm^{-1} to 6000 cm^{-1}, and the right axis indicates the absorbance from 6000 cm^{-1} to 1000 cm^{-1}.

The transient absorption at around 15400 cm^{-1} (649 nm, 1.91 eV) is attributed to photoexcited holes in the $BaTaO_2N$ and Pt-loaded $BaTaO_2N$ samples. The absorption intensity for Pt-loaded $BaTaO_2N$ produced by two-step decoration is clearly higher than that for pristine $BaTaO_2N$ at the same decay time, indicating that the lifetime of photoexcited holes is significantly longer in the Pt-loaded $BaTaO_2N$ sample.

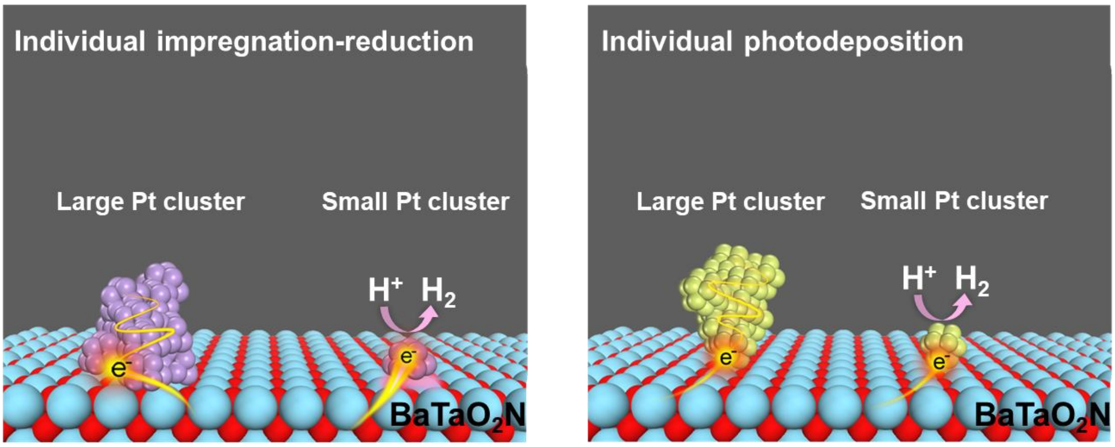

Supplementary Figure S7 | Schematic of Pt cocatalyst deposition on $BaTaO_2N$ by impregnation-reduction or photodeposition method.

The impregnation-reduction method can produce evenly-distributed fine Pt particles for a certain small amount of Pt loading but causes aggregation of Pt particles for higher Pt content. The Pt cocatalyst can still efficiently capture photogenerated electrons from $BaTaO_2N$ because of the intimate contact between the Pt particles and $BaTaO_2N$ photocatalyst, based on the HRTEM and TAS results. However, the Pt-loaded $BaTaO_2N$ exhibited lower photocatalytic H_2-evolution activity than the Pt-loaded $BaTaO_2N$ prepared by the sequential decoration method. This is because aggregation of Pt particles resulted in insufficient catalytic sites, and the large Pt clusters could not efficiently supply the captured electrons for the proton reduction reaction. On the other hand, the Pt cocatalyst is deposited selectively on electron-accumulating sites of $BaTaO_2N$ using the photodeposition method. However, Pt nanoparticles are preferentially localized on some active $BaTaO_2N$ particles to form aggregates and have weak contact with $BaTaO_2N$, which decreases the amount of active catalytic sites and

retards photoexcited electron transfer. Such drawbacks with the impregnation and photodeposition methods reduce the photocatalytic H_2-evolution performance of $BaTaO_2N$.

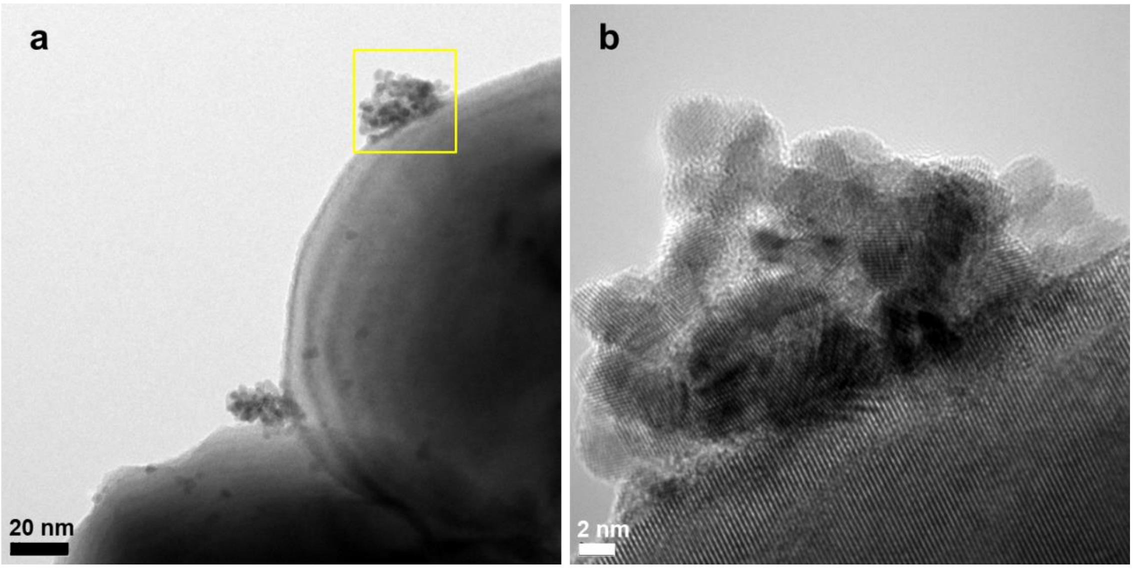

Supplementary Figure S8 | Modification of H_2-treated $BaTaO_2N$ (RbCl) with Pt by photodeposition. **a**,**b**, HRTEM images of $BaTaO_2N$ treated by H_2 gas without introducing Pt, and loaded with 0.2 wt% Pt by photodeposition method, **b** corresponds to the area outlined by a square in **a**.

a

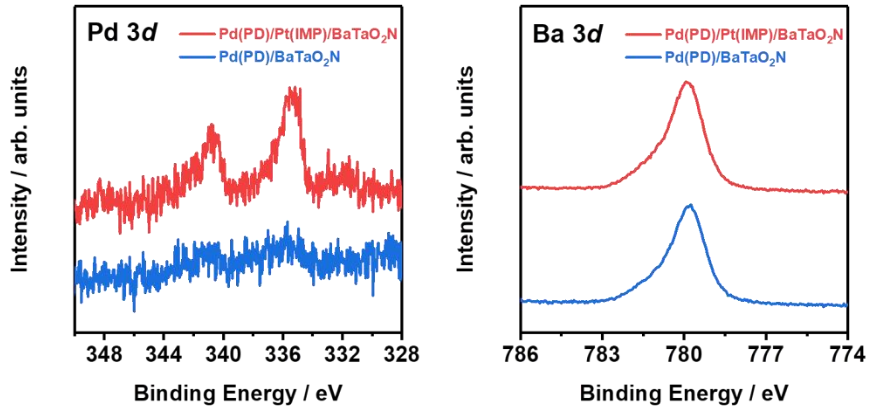

Sample	Atomic ratio between Pd and Ba by XPS Analysis (%)
Pd(PD)/$BaTaO_2N$	0.7
Pd(PD)/Pt(IMP)/$BaTaO_2N$	4.2

b

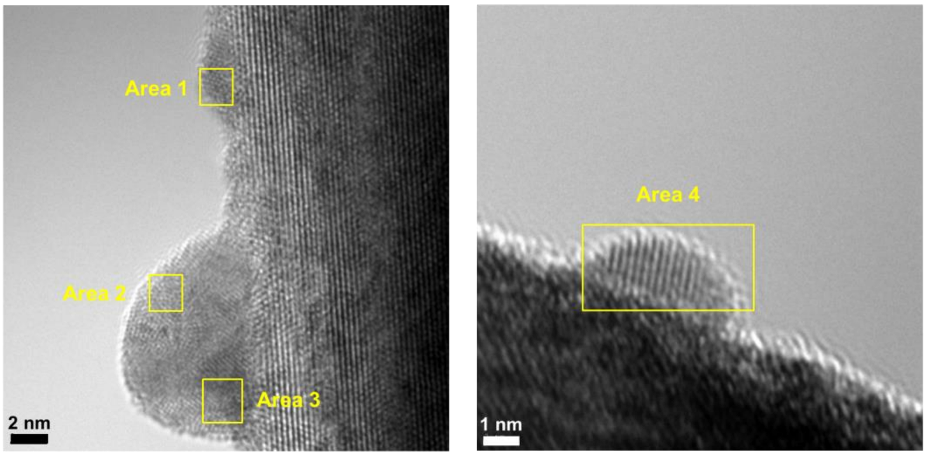

Area	Atomic ratio in the selected area by EDS Analysis (%)	
	Pd	Pt
Area 1	35	65
Area 2	100	0
Area 3	75	25
Area 4	55	45

Supplementary Figure S9 | Photodeposition of Pd on pristine $BaTaO_2N$ (RbCl) and on Pt-impregnated $BaTaO_2N$ (RbCl). a, XPS spectra (Pd 3*d* and Ba 3*d*) acquired from

Electronic Supplementary Information (ESI)

Pd-loaded $BaTaO_2N$ samples. Pd(PD)/$BaTaO_2N$ and Pd(PD)/Pt(IMP)/$BaTaO_2N$ denote Pd loading on pristine $BaTaO_2N$ and Pt-impregnated $BaTaO_2N$, respectively. Photodeposition of Pd on $BaTaO_2N$ was conducted for 1 h. **b**, HRTEM images of the sample after photodeposition of Pd on Pt-impregnated $BaTaO_2N$ for 3 h and EDS elemental analysis results for the selected areas in the HRTEM images.

The XPS results revealed that the atomic ratio of Pd loaded on Pt-impregnated $BaTaO_2N$ was higher than that loaded on pristine $BaTaO_2N$, indicating that the photodeposition of Pd on Pt-impregnated $BaTaO_2N$ occurred more rapidly. This is because the Pt nuclei introduced on $BaTaO_2N$ by impregnation-reduction functioned as electron-accumulation active sites for Pd photodeposition.

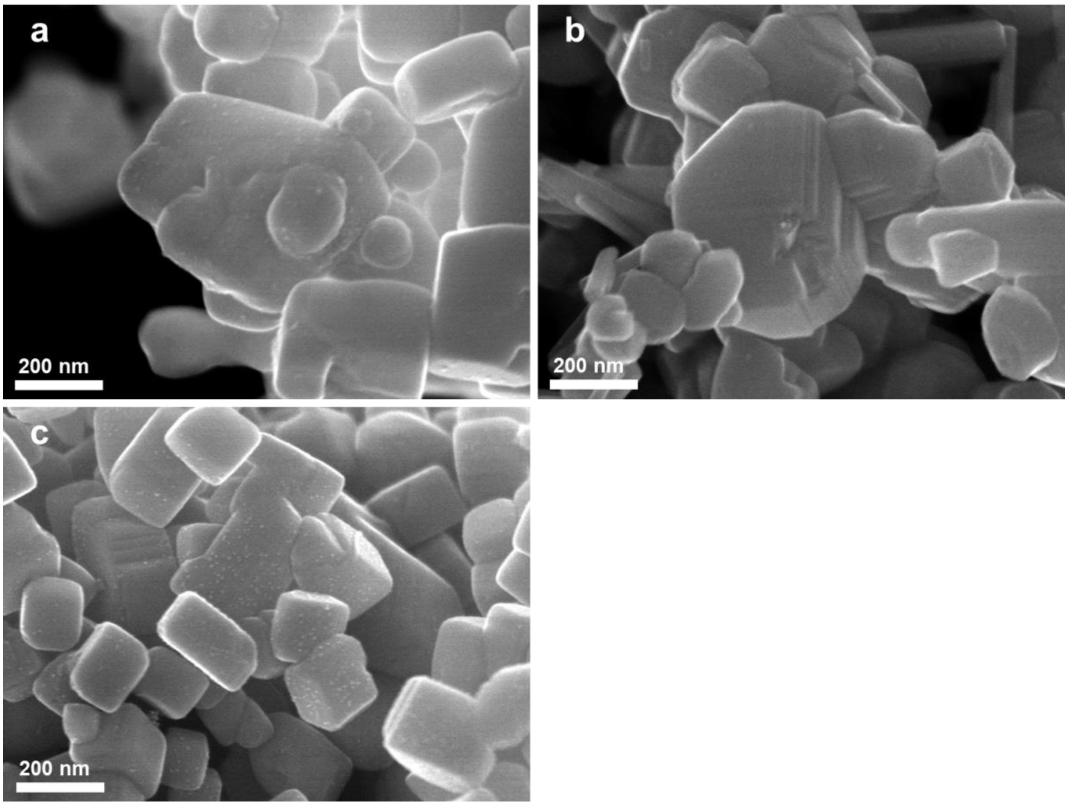

Supplementary Figure S10 | Dispersion of Pt cocatalyst particles on $BaTaO_2N$ photocatalysts. **a**,**b**,**c**, SEM images of Pt-modified $BaTaO_2N$ (NaCl) (**a**), Pt-modified $BaTaO_2N$ (CsCl) (**b**) and Pt-modified $BaTaO_2N$ (KCl) (**c**). Pt content was 0.3 wt% in total by sequential decoration method (0.1 wt% by impregnation-reduction and 0.2 wt% by photodeposition).

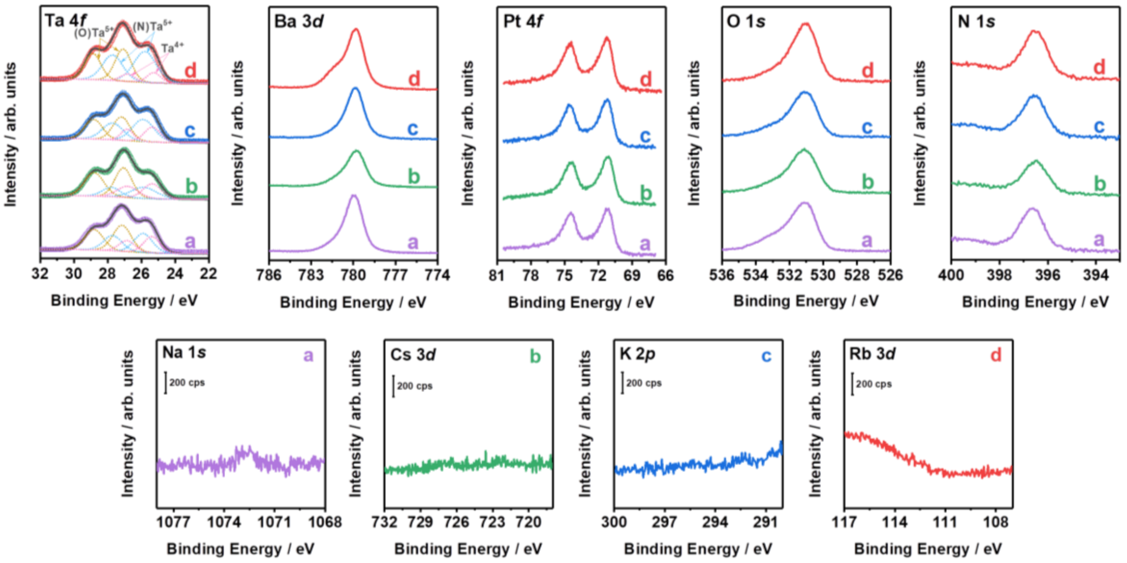

Sample	Atomic ratio between Pt and Ta by XPS Analysis (%)
Pt-modified $BaTaO_2N$ (NaCl)	7.0
Pt-modified $BaTaO_2N$ (CsCl)	7.3
Pt-modified $BaTaO_2N$ (KCl)	7.2
Pt-modified $BaTaO_2N$ (RbCl)	7.4

Supplementary Figure S11 | XPS spectra of different Pt-modified $BaTaO_2N$ photocatalysts. a-d, Ta 4*f*, Ba 3*d*, Pt 4*f*, O 1*s*, N 1*s*, Na 1*s*, Cs 3*d*, K 2*p* and Rb 3*d* spectra acquired from Pt-modified $BaTaO_2N$ (NaCl) (**a**), Pt-modified $BaTaO_2N$ (CsCl) (**b**), Pt-modified $BaTaO_2N$ (KCl) (**c**), Pt-modified $BaTaO_2N$ (RbCl) (**d**). Pt content was 0.3 wt% in total by sequential decoration method (0.1 wt% by impregnation-reduction and 0.2 wt% by photodeposition).

XPS analysis reflects that the incorporation of alkali metal ion in the top surface layers was negligible for $BaTaO_2N$ synthesized with RbCl, KCl or CsCl flux

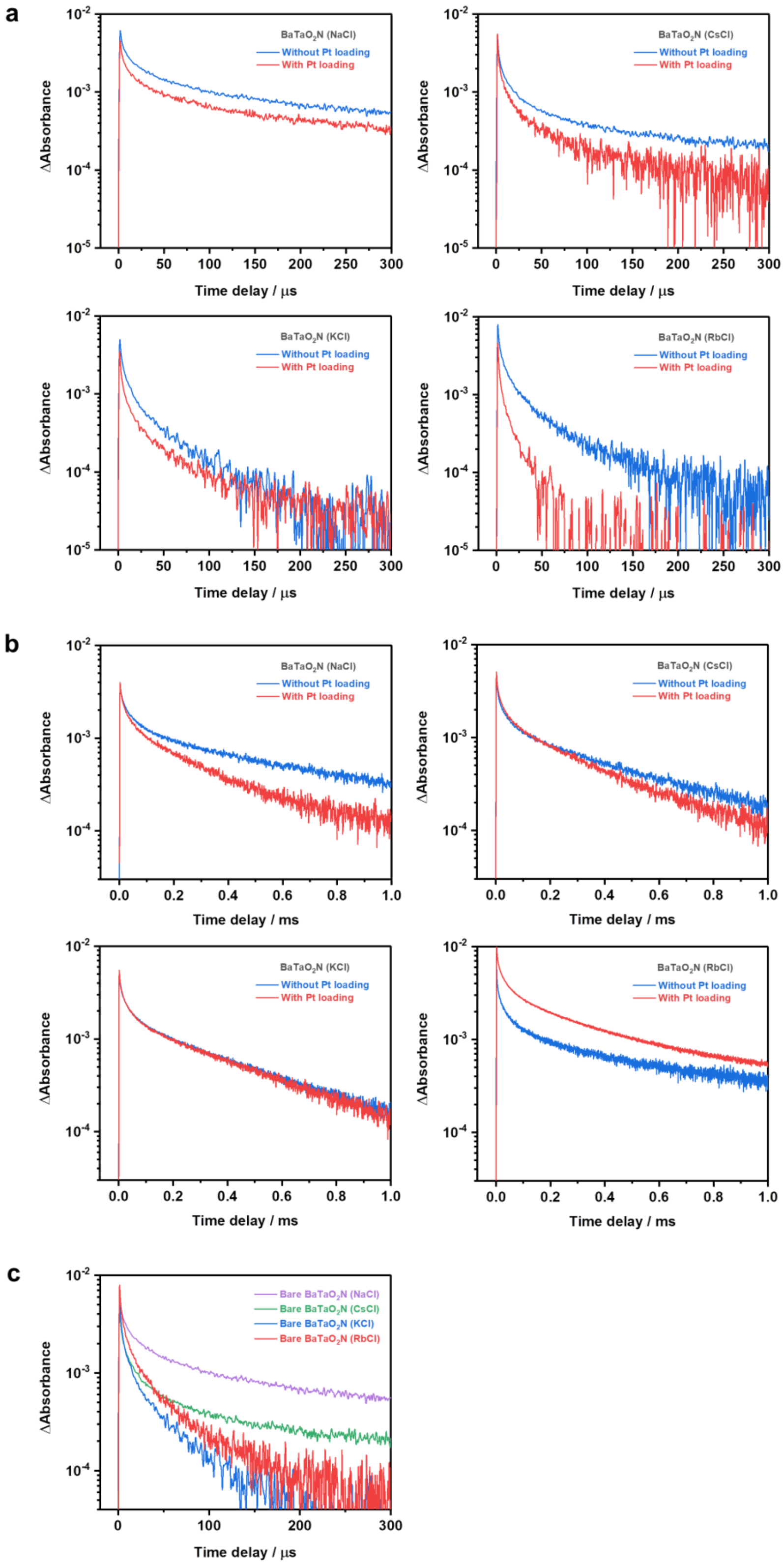

Supplementary Figure S12 | Photoexcited charge dynamics of bare and

Electronic Supplementary Information (ESI)

Pt-modified $BaTaO_2N$ photocatalysts. a, Transient absorption decays corresponding to electron dynamics in bare and Pt-modified $BaTaO_2N$ photocatalysts probed at 11000 cm^{-1} (910 nm, 1.36 eV). **b**, Transient absorption decays corresponding to hole dynamics in bare and Pt-modified $BaTaO_2N$ photocatalysts probed at 15400 cm^{-1} (649 nm, 1.91 eV). **c**, Transient absorption decays corresponding to electron dynamics in bare $BaTaO_2N$ photocatalysts probed at 11000 cm^{-1} (910 nm, 1.36 eV). Pt content was 0.3 wt% in total by sequential decoration method (0.1 wt% by impregnation-reduction and 0.2 wt% by photodeposition).

Comparing the absorption signals of deeply-trapped electrons between bare and Pt-modified $BaTaO_2N$ samples, the largest decrease was observed for $BaTaO_2N$ (RbCl) (Supplementary Fig. 12a). The absorption intensity of photoexcited holes decayed faster for $BaTaO_2N$ (NaCl) and $BaTaO_2N$ (CsCl) after Pt modification (Supplementary Fig. 12b), suggesting that the accelerated electron decay for these two samples was largely due to recombination with holes at defect states. The same hole dynamics for $BaTaO_2N$ (KCl) with and without Pt loading indicate a possible balance in the number of electrons that were captured by Pt and recombined with holes. It is worth noting that the $BaTaO_2N$ (RbCl) photocatalyst exhibited reduced hole decay when the Pt cocatalyst was decorated, indicating a low level of structural defects and mid-gap states in $BaTaO_2N$ (RbCl) acting as recombination centers.

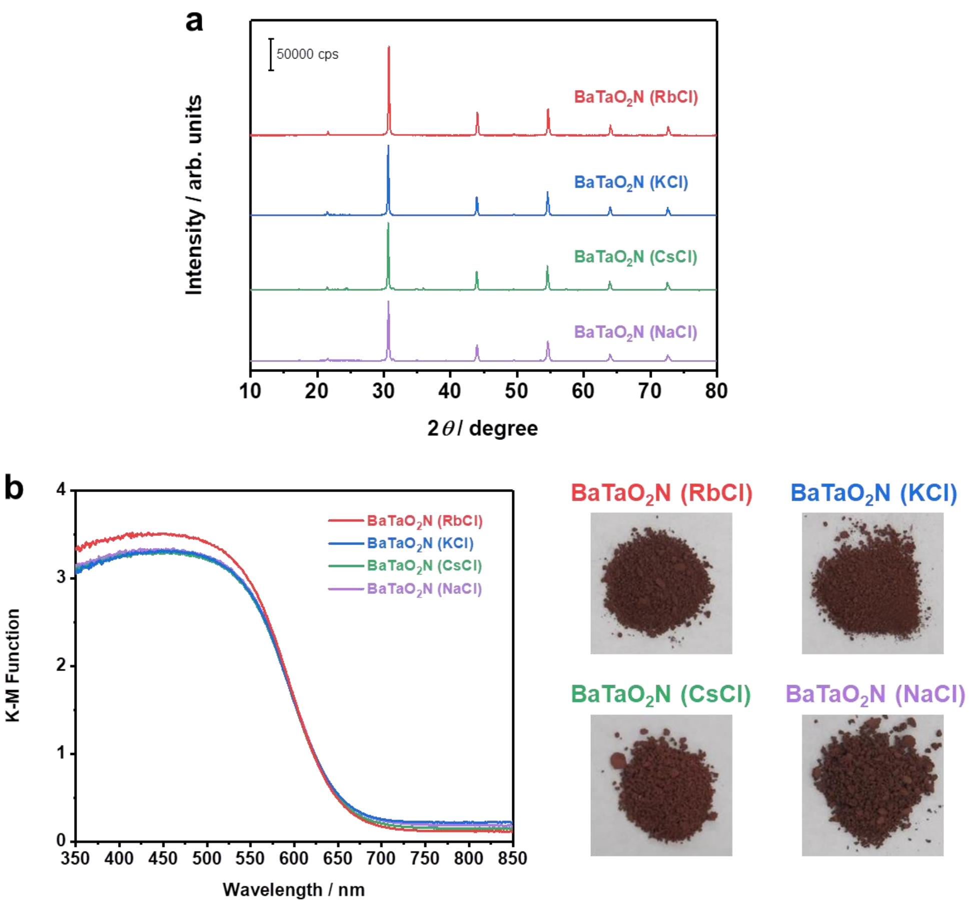

Supplementary Figure S13 | Characterization of bare $BaTaO_2N$ photocatalysts. a, XRD patterns. **b**, UV-vis DRS and optical photos.

XRD patterns show the best crystallized $BaTaO_2N$ was synthesized by RbCl flux-assisted nitridation[20]. These $BaTaO_2N$ materials synthesized with different fluxes exhibited similar brownish-red colour, which agrees well with the same absorption edges at around 650 nm in UV-vis DRS[20,25]. The lowest background absorption for RbCl-assisted $BaTaO_2N$ indicates the minimized defect densities (reduced Ta^{5+} species and anion vacancies) in $BaTaO_2N$ crystal.

Supplementary Table S5 | Atomic ratios of elements in bare $BaTaO_2N$ photocatalysts analysed by ICP-AES and ionic radii of alkali metals.

Sample	Ba (%)	Ta (%)	O (%)	N (%)	Alkali metal (%)	Radius of alkali metal (nm)[a]
$BaTaO_2N$ (NaCl)	19.1	20.1	41.8	17.3	1.6 (Na)	0.139 (Na^+)
$BaTaO_2N$ (KCl)	18.9	19.5	41.5	19.6	0.5 (K)	0.164 (K^+)
$BaTaO_2N$ (RbCl)	20.0	20.4	40.2	19.3	4.6×10^{-2} (Rb)	0.172 (Rb^+)
$BaTaO_2N$ (CsCl)	19.7	20.0	40.9	19.4	2.3×10^{-4} (Cs)	0.188 (Cs^+)

[a] Coordination number was recognized as 12.

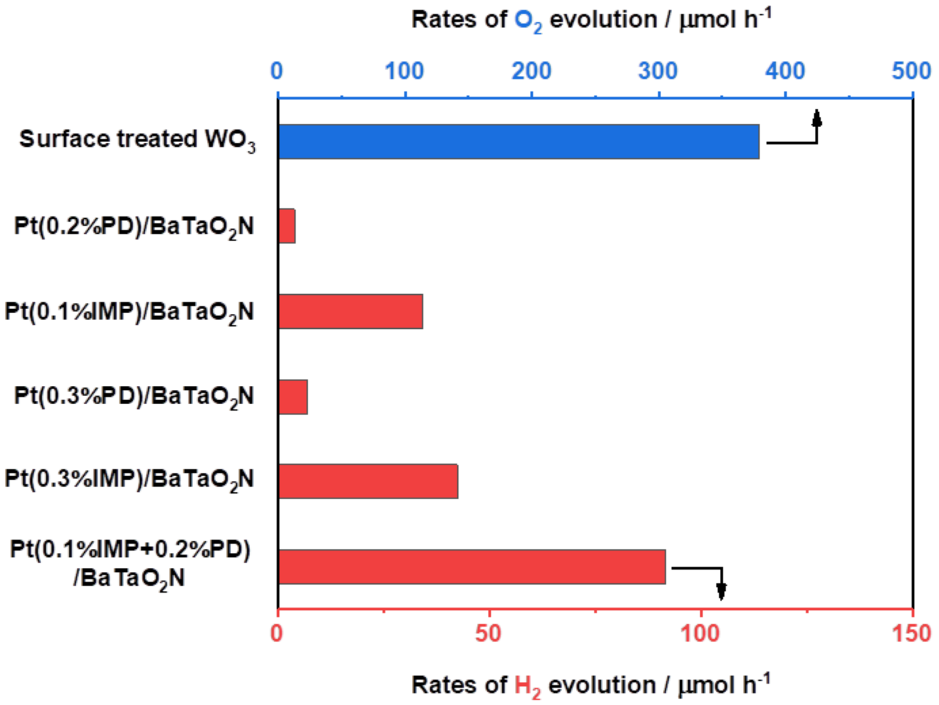

Supplementary Figure S14 | Photocatalytic activity in half-reactions. The H_2 evolution reaction was carried out using Pt-modified $BaTaO_2N$ in the presence of NaI as an electron donor. Conditions: Pt-modified $BaTaO_2N$ (RbCl) photocatalyst, 0.1 g; 5 mM aqueous NaI solution, 150 mL; light source, 300 W Xenon lamp ($\lambda \geq 420$ nm). The O_2 evolution reaction was carried out using surface-treated WO_3 in the presence of $NaIO_3$ as an electron acceptor. Conditions: surface-treated WO_3 photocatalyst, 0.15 g; 20 mM aqueous $NaIO_3$ solution, 150 mL; light source, 300 W Xenon lamp ($\lambda \geq 420$ nm); reaction system, Pyrex top-illuminated vessel connected to closed gas-circulation system without evacuation of gas products.

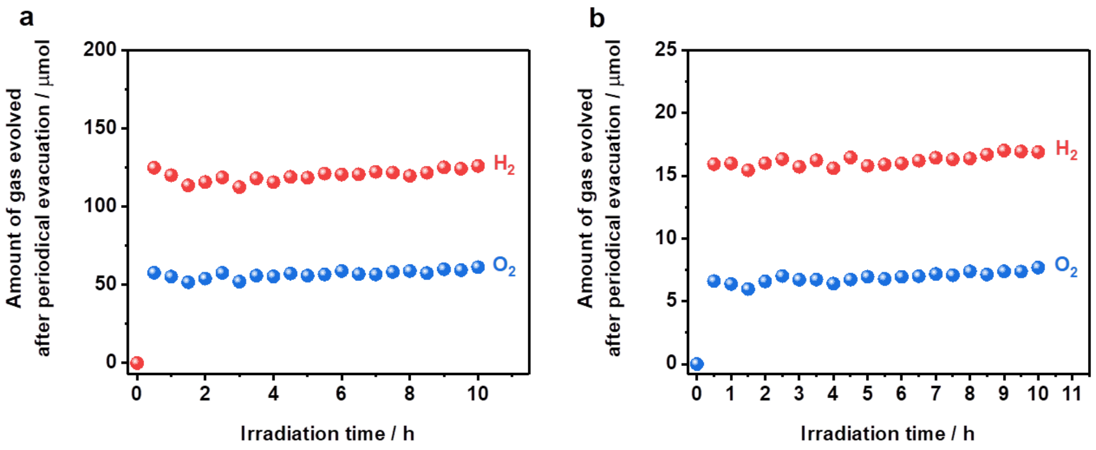

Supplementary Figure S15 | Photocatalytic performance for Z-scheme water splitting. a,**b**, H_2 and O_2 evolution during Z-scheme water splitting reaction with periodic evacuation using Pt(0.1%IMP+0.2%PD)/$BaTaO_2N$ as the HEP, under visible light ($\lambda \geq 420$ nm) (**a**) and simulated sunlight (**b**). Conditions: Pt(0.1%IMP+0.2%PD)/$BaTaO_2N$ (RbCl), 0.1 g; surface-treated WO_3, 0.15 g; 150 mL aqueous NaI solution, 1 mM for **a** and 3 mM for **b**; light source, 300 W Xenon lamp ($\lambda \geq 420$ nm) or solar simulator (AM 1.5G), irradiation area for solar simulator was 7.6 cm^2; reaction system, Pyrex top-illuminated vessel connected to closed gas-circulation system with periodical evacuation of gas products at an interval of 30 min.

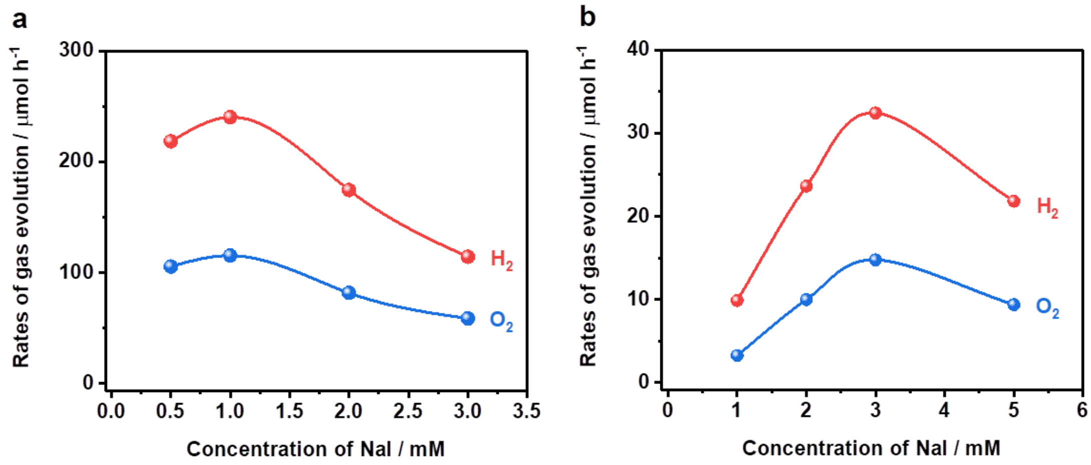

Supplementary Figure S16 | Effect of the redox concentration on Z-scheme water splitting activity. a,**b**, Gas evolution rate as function of concentration of NaI during Z-scheme water splitting over Pt(0.1%IMP+0.2%PD)/$BaTaO_2N$ as the HEP, under visible light ($\lambda \geq$ 420 nm) (**a**) and simulated sunlight (**b**). Conditions: Pt(0.1%IMP+0.2%PD)/$BaTaO_2N$ (RbCl), 0.1 g; surface-treated WO_3, 0.15 g; aqueous NaI solution, 150 mL; light source, 300 W Xenon lamp ($\lambda \geq$ 420 nm) or solar simulator (AM 1.5G), irradiation area for solar simulator was 7.6 cm^2; reaction system, Pyrex top-illuminated vessel connected to closed gas-circulation system with periodical evacuation of gas products.

Electronic Supplementary Information (ESI)

Supplementary Table S6 | Dependence of Z-scheme water splitting under monochromatic light irradiation (λ = 420 nm) on reaction conditions.[a]

Entry	Amount of photocatalyst / g		Concentration of NaI / mM	Rates of gas evolved / μmol h^{-1}		AQY[b] / %
	HEP	OEP		H_2	O_2	
1	0.05	0.10	1	6.0	2.2	1.2
2	0.10	0.15	1	10.7	4.2	2.2
3	0.15	0.25	1	7.6	3.2	1.6
4	0.10	0.15	3	15.7	6.8	3.4
5	0.10	0.15	5	18.6	7.8	4.0
6	0.10	0.15	8	12.9	5.5	2.8

[a] Conditions: Pt(0.1%IMP+0.2%PD)/$BaTaO_2N$ (RbCl) as the HEP; surface-treated WO_3 as the OEP; aqueous NaI solution, 150 mL; light source, 300 W Xenon lamp equipped with a band-pass filter (λ = 420 nm); reaction system, Pyrex top-illuminated vessel connected to closed gas-circulation system with periodical evacuation of gas products.

[b] Estimated from the average of H_2 evolution rates and twice O_2 evolution rates.

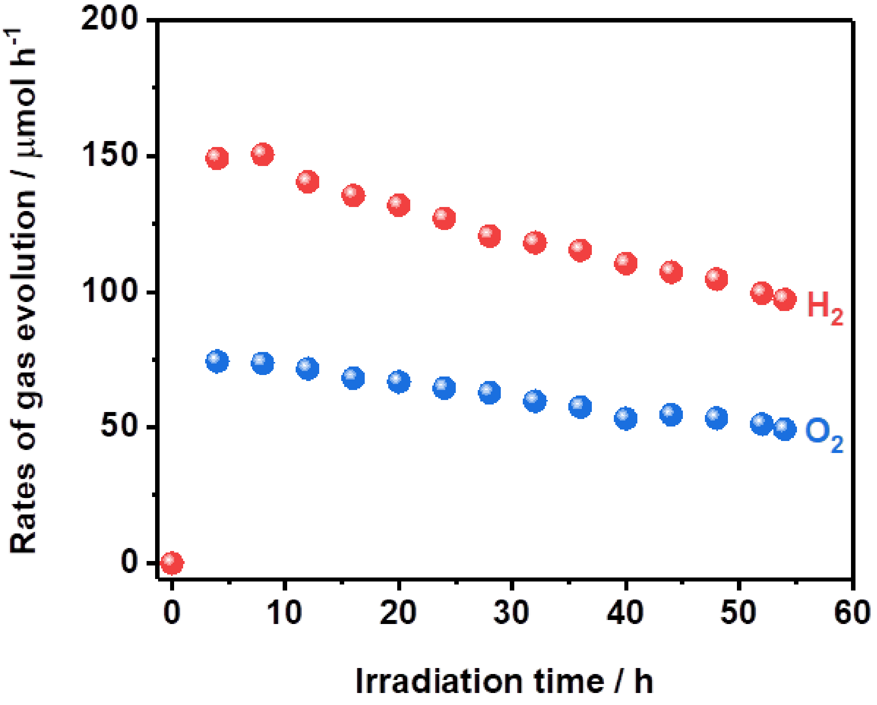

Supplementary Figure S17 | Stability test of Z-scheme water splitting. The reaction was carried out at 6 kPa in the gas flow system with continuous evacuation. Conditions: Pt(0.1%IMP+0.2%PD)/$BaTaO_2N$ (RbCl), 0.1 g; surface-treated WO_3, 0.15 g; 1 mM aqueous NaI solution, 150 mL; light source, 300 W Xenon lamp ($\lambda \geq 420$ nm); reaction system, Pyrex top-illuminated vessel connected to Ar gas-flow system with continuous evacuation of gas products.

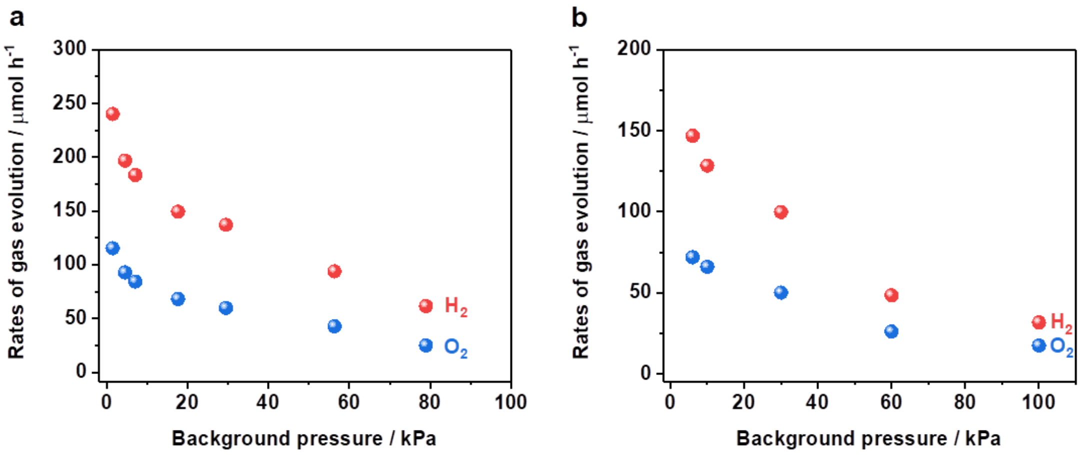

Supplementary Figure S18 | Effect of background pressure on Z-scheme water splitting activity. a,**b**, Gas evolution rate for Z-scheme water splitting as function of background pressure in closed circulation system (**a**) and in gas flow system (**b**). Conditions: Pt(0.1%IMP+0.2%PD)/$BaTaO_2N$ (RbCl), 0.1 g; surface-treated WO_3, 0.15 g; 1 mM aqueous NaI solution, 150 mL; light source, 300 W Xenon lamp ($\lambda \geq 420$ nm); reaction system, Pyrex top-illuminated vessel connected to closed gas-circulation system with introduction of Ar gas and without evacuation of gas products for **a**, connected to gas-flow system with continuous Ar gas feed and continuous evacuation of gas products for **b**.

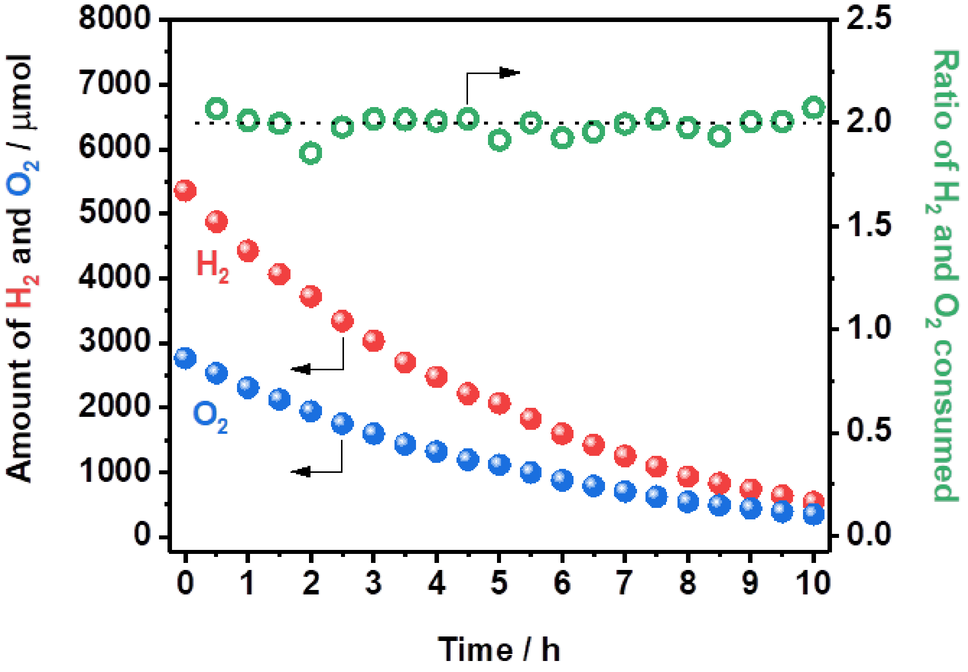

Supplementary Figure S19 | Water formation from H_2 and O_2 in darkness. Conditions: Pt(0.1%IMP+0.2%PD)/$BaTaO_2N$ (RbCl), 0.1 g; surface-treated WO_3, 0.15 g; 1 mM aqueous NaI solution, 150 mL.

This experiment was carried out in a closed circulation system containing a stoichiometric mixture of H_2 and O_2 gases (5360 µmol for H_2 and 2760 µmol for O_2). The decreases in H_2 and O_2 were in the stoichiometric ratio of 2 to 1, indicating a water formation reaction. Nearly 90% of the H_2 and O_2 were consumed in 10 h.

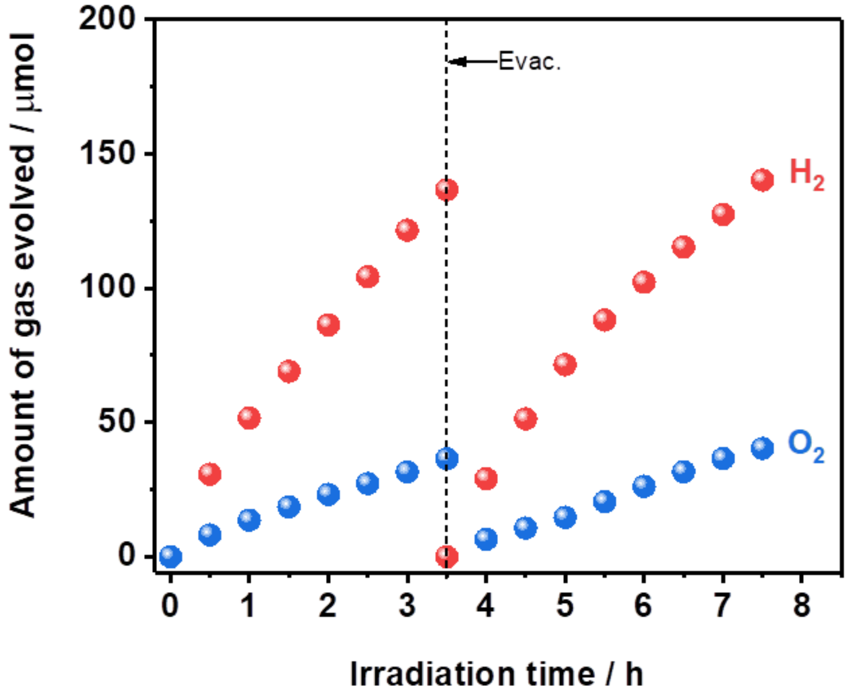

Supplementary Figure S20 | Time course of Z-scheme water splitting. The Z-scheme water splitting system was constructed with Pt(0.1%IMP+0.2%PD)/$BaTaO_2N$ as the HEP, and untreated WO_3 as the OEP. Conditions: Pt(0.1%IMP+0.2%PD)/$BaTaO_2N$ (RbCl), 0.1 g; untreated WO_3, 0.1 g; 5 mM aqueous NaI solution, 150 mL; light source, 300 W Xenon lamp ($\lambda \geq 420$ nm); reaction system, Pyrex top-illuminated vessel connected to closed gas-circulation system with intermediate evacuation of gas products at 3.5 h illumination.

In the redox-mediated Z-scheme water splitting system, reverse reactions including the reduction of oxidized mediators on the HEP and the oxidation of reduced mediators on the OEP compete with the water splitting reaction and reduce the total activity. When I^-

anions were used as an electron mediator, they were oxidized on Pt-modified $BaTaO_2N$ to form IO_3^- and I_3^- species[8,14]. The adsorption and reduction of IO_3^- and I_3^- species on the untreated WO_3 were less efficient than on the surface-treated WO_3[26], and the reduction of remaining IO_3^- and I_3^- species competed with the H_2 evolution reaction on Pt-modified $BaTaO_2N$. Therefore, both the H_2 and O_2 evolution rates were low and the stoichiometric ratio was not achieved when untreated WO_3 was used as the OEP. The use of surface-treated WO_3 suppresses the reverse reaction involving redox mediators on Pt-modified $BaTaO_2N$ and helps improve the intrinsic performance of $BaTaO_2N$ in the Z-scheme water splitting system.

Electronic Supplementary Information (ESI)

References

1. Sayama, K., Mukasa, K., Abe, R., Abe, Y. & Arakawa, H. Stoichiometric water splitting into H_2 and O_2 using a mixture of two different photocatalysts and an IO_3^-/I^- shuttle redox mediator under visible light irradiation. *Chem. Commun.* 2416−2417 (2001).
2. Kato, H., Sasaki, Y., Shirakura, N. & Kudo, A. Synthesis of highly active rhodium-doped $SrTiO_3$ powders in Z-scheme systems for visible-light-driven photocatalytic overall water splitting. *J. Mater. Chem. A* **1**, 12327−12333 (2013).
3. Wang, Q. *et al*. Scalable water splitting on particulate photocatalyst sheets with a solar-to-hydrogen energy conversion efficiency exceeding 1%. *Nat. Mater.* **15**, 611−615 (2016).
4. Abe, R., Takata, T., Sugihara, H. & Domen, K. Photocatalytic overall water splitting under visible light by TaON and WO_3 with an IO_3^-/I^- shuttle redox mediator. *Chem. Commun.* 3829−3831 (2005).
5. Maeda, K., Higashi, M., Lu, D., Abe, R. & Domen, K. Efficient nonsacrificial water splitting through two-step photoexcitation by visible light using a modified oxynitride as a hydrogen evolution photocatalyst. *J. Am. Chem. Soc.* **132**, 5858−5868 (2010).
6. Chen, S. *et al*. Efficient visible-light-driven Z-scheme overall water splitting using a $MgTa_2O_{6-x}N_y$/TaON heterostructure photocatalyst for H_2 evolution. *Angew. Chem. Int. Ed.* **54**, 8498−8501 (2015).
7. Qi, Y. *et al*. Redox-based visible-light-driven Z-scheme overall water splitting with apparent quantum efficiency exceeding 10%. *Joule* **2**, 2393−2402 (2018).

8. Ma, G. *et al.* Visible light-driven Z-scheme water splitting using oxysulfide H_2 evolution photocatalysts. *J. Phys. Chem. Lett.* **7**, 3892−3896 (2016).
9. Sun, S. *et al.* Efficient redox-mediator-free Z-scheme water splitting employing oxysulfide photocatalysts under visible light. *ACS Catal.* **8**, 1690−1696 (2018).
10. Iwase, A. *et al.* Water splitting and CO_2 reduction under visible light irradiation using Z-scheme systems consisting of metal sulfides, CoO_x-loaded $BiVO_4$, and a reduced graphene oxide electron mediator. *J. Am. Chem. Soc.* **138**, 10260−10264 (2016).
11. Chen, S. *et al.* Metal selenide photocatalysts for visible-light-driven Z-scheme pure water splitting. *J. Mater. Chem. A* **7**, 7415−7422 (2019).
12. Abe, R., Shinmei, K., Koumura, N., Hara, K. & Ohtani, B. Visible-light-induced water splitting based on two-step photoexcitation between dye-sensitized layered niobate and tungsten oxide photocatalysts in the presence of a triiodide/iodide shuttle redox mediator. *J. Am. Chem. Soc.* **135**, 16872−16884 (2013).
13. Higashi, M., Abe, R., Takata, T. & Domen, K. Photocatalytic overall water splitting under visible light using $ATaO_2N$ (A = Ca, Sr, Ba) and WO_3 in a IO_3^-/I^- shuttle redox mediated system. *Chem. Mater.* **21**, 1543−1549 (2009).
14. Maeda, K., Lu, D. & Domen, K. Solar-driven Z-scheme water splitting using modified $BaZrO_3$-$BaTaO_2N$ solid solutions as photocatalysts. *ACS Catal.* **3**, 1026−1033 (2013).
15. Qi, Y. *et al.* Achievement of visible-light-driven Z-scheme overall water splitting using barium-modified Ta_3N_5 as a H_2-evolving photocatalyst. *Chem. Sci.* **8**, 437−443 (2017).

16. Dong, B. *et al*. Heterostructure of 1D Ta_3N_5 nanorod/$BaTaO_2N$ nanoparticle fabricated by a one-step ammonia thermal route for remarkably promoted solar hydrogen production. *Adv. Mater.* **31**, 1808185 (2019).
17. Dong, B. *et al*. Synthesis of $BaTaO_2N$ oxynitride from Ba-rich oxide precursor for construction of visible-light-driven Z-scheme overall water splitting. *Dalton Trans.* **46**, 10707−10713 (2017).
18. Matoba, T., Maeda, K. & Domen, K. Activation of $BaTaO_2N$ photocatalyst for enhanced non-sacrificial hydrogen evolution from water under visible light by forming a solid solution with $BaZrO_3$. *Chem. Eur. J.* **17**, 14731−14735 (2011).
19. Maeda, K. & Domen, K. Water oxidation using a particulate $BaZrO_3$-$BaTaO_2N$ solid-solution photocatalyst that operates under a wide range of visible light. *Angew. Chem. Int. Ed.* **51**, 9865−9869 (2012).
20. Luo, Y. *et al*. Fabrication of single-crystalline $BaTaO_2N$ from chloride fluxes for photocatalytic H_2 evolution under visible light. *Cryst. Growth Des.* **20**, 255−261 (2020).
21. Zhang, H., Wei, S. & Xu, X. Mg modified $BaTaO_2N$ as an efficient visible-light-active photocatalyst for water oxidation. *J. Catal.* **383**, 135−143 (2020).
22. Jadhav, S. *et al*. Efficient photocatalytic oxygen evolution using $BaTaO_2N$ obtained from nitridation of perovskite-type oxide. *J. Mater. Chem. A* **8**, 1127−1130 (2020).
23. Momma, K., & Izumi, F. VESTA 3 for three-dimensional visualization of crystal, volumetric and morphology data. *J. Appl. Crystallogr.* **44**, 1272−1276 (2011).
24. Cahen, D. & Lester, J. E. Mixed and partial oxidation states. Photoelectron spectroscopic evidence. *Chem. Phys. Lett.* **18**, 108−111 (1973).

25. Luo, Y. *et al*. Construction of spatial charge separation facets on $BaTaO_2N$ crystals by flux growth approach for visible-light-driven H_2 production. *ACS Appl. Mater. Interfaces* **11**, 22264−22271 (2019).

26. Miseki, Y., Fujiyoshi, S., Gunji, T. & Sayama, K. Photocatalytic water splitting under visible light utilizing I_3^-/I^- and IO_3^-/I^- redox mediators by Z-scheme system using surface treated PtO_x/WO_3 as O_2 evolution photocatalyst. *Catal. Sci. Technol.* **3**, 1750−1756 (2013).
